# Socioeconomic Deprivation, Adverse Childhood Experiences and Medical Disorders in Adulthood: Mechanisms and Associations

**DOI:** 10.1007/s12035-019-1498-1

**Published:** 2019-01-26

**Authors:** Gerwyn Morris, Michael Berk, Michael Maes, André F. Carvalho, Basant K. Puri

**Affiliations:** 1 IMPACT Strategic Research Centre, School of Medicine, Deakin University, Barwon Health, P.O. Box 291, Geelong, Victoria Australia; 2 Department of Psychiatry, Level 1 North, Main Block, Royal Melbourne Hospital, University of Melbourne, Parkville, Victoria Australia; 3 Florey Institute for Neuroscience and Mental Health, University of Melbourne, Kenneth Myer Building, 30 Royal Parade, Parkville, Victoria Australia; 4 Orygen, The National Centre of Excellence in Youth Mental Health, 35 Poplar Rd, Parkville, Victoria Australia; 5 Department of Psychiatry, Chulalongkorn University, Bangkok, Thailand; 6 Department of Psychiatry, Faculty of Medicine, University of Toronto, Toronto, ON Canada; 7 Centre for Addiction & Mental Health (CAMH), Toronto, ON Canada; 8 Department of Medicine, Hammersmith Hospital, Imperial College London, London, UK

**Keywords:** Adverse childhood experiences, Gene expression, Hypothalamic–pituitary–adrenal axis, Immune system, Mitochondria, Socioeconomic deprivation

## Abstract

Severe socioeconomic deprivation (SED) and adverse childhood experiences (ACE) are significantly associated with the development in adulthood of (i) enhanced inflammatory status and/or hypothalamic–pituitary–adrenal (HPA) axis dysfunction and (ii) neurological, neuroprogressive, inflammatory and autoimmune diseases. The mechanisms by which these associations take place are detailed. The two sets of consequences are themselves strongly associated, with the first set likely contributing to the second. Mechanisms enabling bidirectional communication between the immune system and the brain are described, including complex signalling pathways facilitated by factors at the level of immune cells. Also detailed are mechanisms underpinning the association between SED, ACE and the genesis of peripheral inflammation, including epigenetic changes to immune system-related gene expression. The duration and magnitude of inflammatory responses can be influenced by genetic factors, including single nucleotide polymorphisms, and by epigenetic factors, whereby pro-inflammatory cytokines, reactive oxygen species, reactive nitrogen species and nuclear factor-κB affect gene DNA methylation and histone acetylation and also induce several microRNAs including miR-155, miR-181b-1 and miR-146a. Adult HPA axis activity is regulated by (i) genetic factors, such as glucocorticoid receptor polymorphisms; (ii) epigenetic factors affecting glucocorticoid receptor function or expression, including the methylation status of alternative promoter regions of *NR3C1* and the methylation of *FKBP5* and *HSD11β2*; (iii) chronic inflammation and chronic nitrosative and oxidative stress. Finally, it is shown how severe psychological stress adversely affects mitochondrial structure and functioning and is associated with changes in brain mitochondrial DNA copy number and transcription; mitochondria can act as couriers of childhood stress into adulthood.

## Introduction

There is a large body of research demonstrating the existence of a significant association between severe socioeconomic deprivation (SED) and adverse childhood experiences (ACE) and the development of enhanced inflammatory status and/or hypothalamic–pituitary–adrenal (HPA) axis dysfunction in adulthood [1–3]. There are also parallel data demonstrating a positive association between SED and ACE in childhood and the subsequent development of a wide range of neurological, neuroprogressive, inflammatory and autoimmune diseases [1]. The latter association would seem unsurprising given a wealth of data implicating a role for increased levels of peripheral inflammation and/or HPA axis dysfunction in the pathogenesis and pathophysiology of several of these illnesses [4–6]. Indeed, some form of causal relationship between the inflammatory burden and HPA axis transferred from childhood in the aetiology of these illnesses is suspected [2, 7]. However, such HPA axis dysfunction and increased inflammatory burden is seen in adults exposed to SED and ACE and adverse experiences in childhood are seen in adults with no discernible evidence of pathology [8–10]. This suggests that other factors determine whether these experiences lead to overt pathology or not.

One concept invoked to explain these observations at least in part is the phenomenon of psychological resilience. Readers interested in an examination of the evidence for and against this explanation are invited to consult recent papers by [11, 12]. Another heuristic invoked in an attempt to explain this central question is the concept of allostatic load and the failure of adaptive mechanisms (allostasis) leading to allostatic overload and the subsequent development of pathology in adulthood or adolescence [13]. Over a lifetime, allostasis and allostatic loads (or pathophysiology) epigenetically affect both central and peripheral processes: stressful phenomena, including environmental stressors, major life events, abuse and trauma, can affect the development of stress susceptibility and lead to epigenetic changes in central neural circuitry and functioning; in turn, this can feed into perceived stress, in terms of vigilance and helplessness [13]. This, in turn, is associated with behavioural and physiological responses which mediate allostasis, leading to adaptation; as a result of, for example, repeated stress or dysregulated stress responses (that is, allostatic load), it has been argued that this can lead to pathophysiology [13].

Recent data suggest that the extent of allostatic load appears to be determined by defects in the responses of the autonomic nervous system and the HPA axis as well as increasing levels of lipids and glucose and the extent of inflammation, nitrosative and oxidative stress and mitochondrial dysfunction in any given individual [14, 15] (reviewed in [16]).

Genetic and epigenetic elements influence all the elements comprising allostatic load as discussed above. For example the weight of evidence indicates that polymorphisms in cytokine and/or Toll-like receptor (TLR) genes and other variations in the genome and epigenome may influence the magnitude and duration of inflammatory responses to cellular stimuli and the subsequent levels of oxidative stress [17–20]. Abnormalities in the epigenetic regulation of the activity of these and other genes can have similar consequences [21]. Epigenetic and genetic factors also influence the performance of mitochondria [22, 23]. Mitochondrial function also affects lipid and glucose levels in any given individual (reviewed in [24, 25]). Mitochondrial dysfunction alone and in combination with raised lipid and glucose levels can also add to the burden of inflammation and oxidative stress in the periphery and the brain [26–28]. Predictably, the response of the neuroendocrine system and the HPA axis to severe and or prolonged psychosocial stress is also under genetic and epigenetic influences [13, 29, 30] and the abnormal performance of both systems can make an independent contribution to whole body increases in inflammation oxidative stress and indeed mitochondrial dysfunction [1, 31]. It should also be noted that increased levels of inflammation, oxidative stress and mitochondrial dysfunction can conspire to provoke epigenetic dysregulation via altered levels of DNA methylation, histone modifications (such as acetylation) and microRNA (miRNA) synthesis [23, 32–34].

These are important observations, as high levels of inflammation and oxidative stress can induce tissue damage and the formation of damage-associated molecular patterns (DAMPs) which can engage with pattern recognition receptors (PRRs) on antigen-presenting cells (APCs) with the production of pro-inflammatory cytokines (PICs) and reactive oxygen species (ROS), thus maintaining and indeed amplifying a chronic state of peripheral inflammation, nitrosative and oxidative stress and mitochondrial dysfunction [35, 36]. There is also evidence that such a state in the periphery can lead to the activation of microglia and astrocytes in the brain leading to the development of chronic neuroinflammation with increased levels of ROS in the brain together with a stream of other neurotoxic consequences such as disturbance to neurotransmission and neuron to glial cell communication known to contribute to the development of neuropsychiatric and neurodegenerative conditions [6, 37]. It should also be noted that activation of these glial cells and the neuropathology stemming from such activation can be induced by stress-related increase in glucocorticoid (GC) levels in the CNS even in the absence of peripheral inflammation [38–40]. This level of chronic inflammation can inhibit HPA axis activity, which may also be inhibited more directly by GC resistance, including glucocorticoid receptor (GR) methylation [30, 41].

When considered as a whole, there is considerable evidence in support of the proposition that the allostatic load experienced during childhood can inhibit allostasis leading to the development of allostatic overload in adulthood in the context of genetic and or epigenetic vulnerability. There is also growing evidence that the “unholy trinity” of mitochondrial dysfunction, nitrosative and oxidative stress and inflammation may well be the most significant contributors to allostatic load [14]. A detailed consideration of the mechanisms underpinning the origin of this triad in at least some children subjected to such adverse environmental conditions and their overall detrimental influence on the other mediators of allostasis and the persistence of such abnormalities into adulthood is the main focus of this paper.

Much of the early research in this area has been focused on the bidirectional interaction between peripheral inflammation resulting from PIC release by APCs in the periphery as part of an evolutionary response to environmental stressors and the neuroendocrine system. Hence, we will consider such research in the first section before going on to consider more recent findings regarding the importance of oxidative stress in the brain and mitochondrial dysfunction. First, however, it seems appropriate to review the quality and quantity of evidence demonstrating an association between the experience of SED and other ACE in childhood and the development of neuropsychiatric, neurodegenerative and autoimmune illnesses in adulthood.

## Childhood SED and/or ACE and the Development of Illnesses in Adulthood

### Overview

There is a large and accumulating body of evidence demonstrating an association between adverse socioeconomic or caregiving experiences in childhood and increased risk of morbidity and mortality from non-communicable chronic health problems in adulthood [42–44], including rheumatoid arthritis (RA), systemic lupus erythematosus (SLE), Sjögren’s syndrome, cardiovascular disease, diabetes, hypertension, chronic fatigue syndrome (CFS) fibromyalgia, Alzheimer’s disease (AD), Parkinson’s disease, amyotrophic lateral sclerosis (ALS), major depressive disorder (MDD), anxiety, cancer and all causes of premature mortality [43, 45–51]. It is also noteworthy that many authors have reported that such an association persists even in the face of significant increases in the quality of life experienced by individuals during adolescence and adulthood [52, 53].

### SED, ACE and the Development of Chronic Inflammation

Several, mainly cross-sectional, studies have demonstrated that environmental and socioeconomic adversity in childhood is associated with significantly increased levels of systemic inflammation in both childhood [54–56] and adulthood [9, 31, 57–59]. It seems worthy of note that lower education and income appear to be particular factors associated with the development of an inflammatory burden in adulthood [8, 57, 60–62]. Such inflammatory activity is evidenced by significantly elevated levels of the inflammatory markers C-reactive protein (CRP) [63, 64], IL-6 [60, 61, 65], TNF-α [57, 65], IL-2 [57, 65] and NF-κB [66, 67]. Unsurprisingly, recent studies using a life course approach identified inflammation markers as at least in part explaining social differences in health [68, 69]. Additional studies revealed that both early life and adult adverse socioeconomic circumstances have the potential to alter inflammation status [70, 71]. It should be emphasised however that the results in this area are heavily influenced by study design as we will now move on to consider.

### Study Design Effects Regarding SED, ACE and Increased Risk of Adult Illness

Socioeconomic status (SES) is a mediator, marker and moderator of diverse other operative variables [72]. Prospective studies investigating correlations between low SES in childhood and increases in single markers of inflammation in adulthood have reported weakly significant or non-significant associations following adjustment for other variables such as ethnicity, adult SES, body mass index or educational status [49, 72–74]. However, a longitudinal study performed by Castagne and fellow workers examining the levels of a wide range of cytokines and chemokines using multiplex technology reported a significant association between low childhood SES and adult inflammatory status, although the increases in cytokine and chemokine levels compared with adults who had a benign childhood economic status were modest [2]. This association has been confirmed in the prospective Brazilian Longitudinal Study of Adult Health (ELSA-Brasil) involving 13,371 civil servants [75]. These authors reported a linear increase in the inflammatory marker CRP with an increasing number of adverse socioeconomic circumstances throughout the individual’s life course [75]. These findings were replicated in the Jerusalem Perinatal Family Follow-Up Study by [76] involving follow-up examinations of 1,132 offspring of all the births in Jerusalem between 1974 and 1976. The authors established that adult and childhood economic status were independent contributors to adult inflammatory status [76]. A similar pattern emerged from studies investigating the relationship between other childhood stressors such as maltreatment and adult inflammatory status. A meta-analysis of 60 retrospective cross-sectional studies examining this phenomenon involving 16,870 participants conducted by Baumeister and fellow workers concluded that childhood mistreatment was significantly associated with a modest increase in CRP, TNF-α and IL-6 levels, although the association with increased CRP was stronger than for the cytokines [77]. This supported the conclusions of an earlier prospective study conducted over 20 years which demonstrated that childhood maltreatment was independently associated with modestly increased levels of CRP, IL-6 and TNF-α in adulthood [63].

## SED, ACE and the Development of HPA Axis Dysfunction in Adulthood

### Evidence Associating SED, ACE and Dysfunction of HPA Axis Activity

There is a considerable body of evidence indicating that children from disadvantaged backgrounds experiencing environmental stressors such as low SES and poor housing conditions are significantly more likely to display evidence of persistent HPA axis dysregulation [78–81]. In addition, the weight of evidence suggests that the aggregate effect of SED and ACE, rather than the impact of individual risks, is deterministic of pathological outcomes (reviewed in [3]). A comprehensive meta-analysis of 26 cross-sectional studies examining the association between SES and cortisol levels conducted by Dowd and fellow workers concluded that a slightly blunted pattern of diurnal cortisol secretion in association with lower SES was the most consistent observation, although overall there was no consistent association between SES and cortisol levels [82]. Several prospective studies have examined longitudinal changes in HPA axis function in consort with changes in SES over time and have generally reported a negative association between SES and concentrations of cortisol present in hair samples [83–85]. There is a growing consensus that the measurement of hair cortisol levels is the most appropriate method for measuring aggregate concentrations of this molecule over time [86].

### HPA Axis Dysfunction and the Development of Adult Diseases

HPA axis hypofunction in particular is found in illnesses such as RA [87–89], Sjögren’s syndrome [90, 91], CFS [92], SLE [93, 94], multiple sclerosis (MS) [95, 96] and MDD [97]. Blunted or deregulated HPA axis activity is also seen in people suffering from chronic pain (reviewed in [98]), chronic fatigue [99] and chronic insomnia [100]. There is also a large body of evidence supporting the concept that severe early-life experiences result in HPA axis dysfunction in adulthood and the development of peripheral inflammation [77]. The HPA axis is also a powerful modulator of inflammatory activity and is in turn modulated by inflammatory processes [7, 30, 101]. The mechanisms underpinning such a bidirectional relationship go some way to explaining how HPA axis dysfunction might contribute to the development of inflammatory diseases in adulthood and hence will be the subject of the next section of the paper.

## Mechanisms Enabling Bidirectional Communication Between the Immune System and the Brain

### Overview

Bidirectional communication between the immune system and the CNS involving highly complex signalling pathways plays an indispensable role in restraining immune and inflammatory responses in physiological and pathological conditions [102]. This communication is facilitated at the level of immune cells by several mechanisms, such as the expression of surface receptors for several different neurotransmitters, allowing the brain to modulate the immune response [103, 104]) and the secretion of opioids and immunomodulatory catecholamines such as noradrenaline (norepinephrine) and adrenaline (epinephrine) and by acetylcholine [103, 105]. Peripheral mononuclear blood cells (PMBCs) also express receptors for a wide array of ligands such as corticosteroids, prolactin, insulin, somatostatin, growth hormone (GH), testosterone, oestrogen, ghrelin, leptin, opioids, neuropeptide Y and vasoactive intestinal peptide (VIP) [106].

**Fig. 1:**
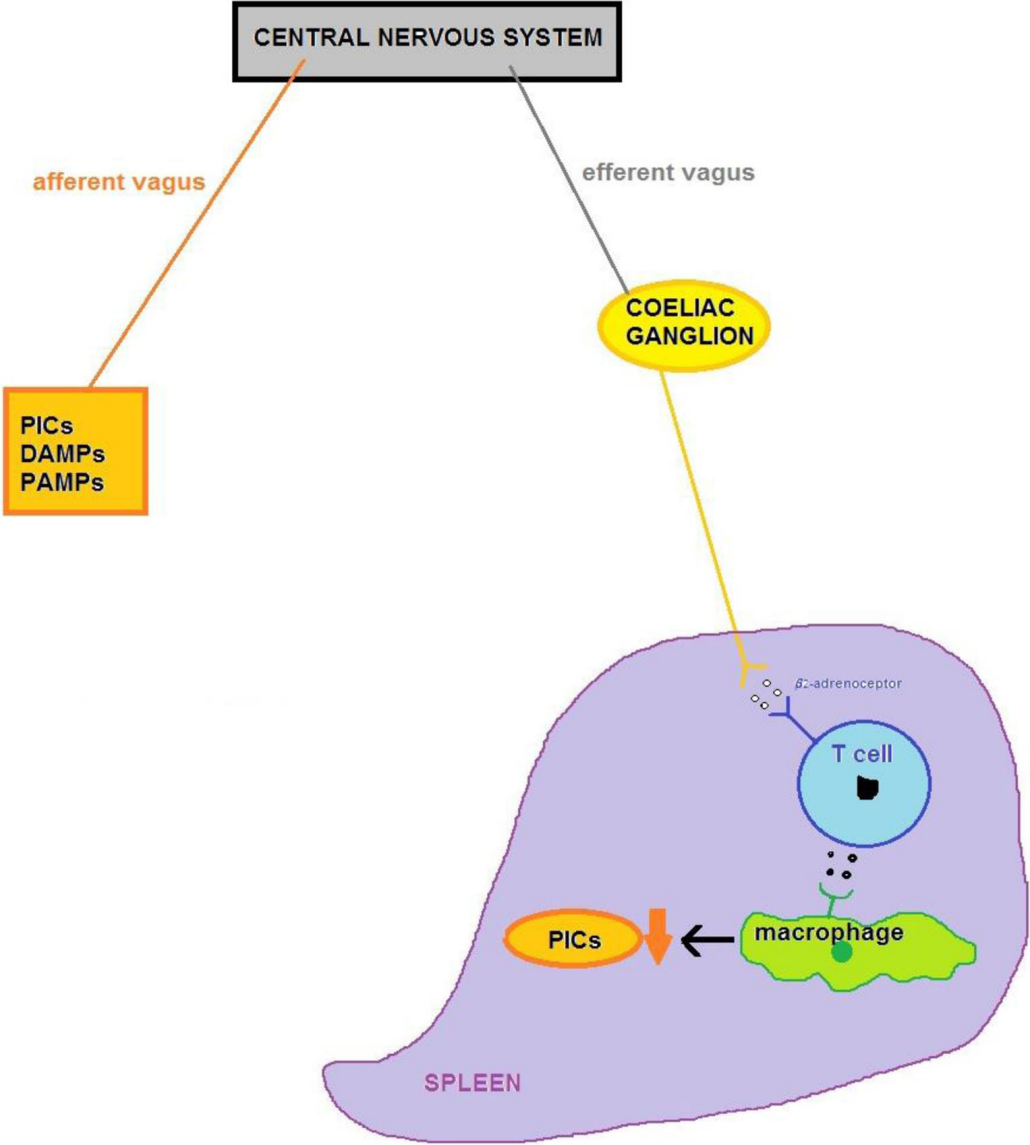
The circuit of the anti-inflammatory reflex is enabled by signals carried by the afferent and efferent branches of the vagus nerve. The afferent branch is activated in response to the presence of PICs, DAMPs and PAMPs in peripheral tissues. The efferent signal is communicated via the coeliac ganglion and activates splenic adrenergic neurones inducing the release of noradrenaline (norepinephrine) near acetylcholine secreting T cells. Released acetylcholine then transverses the marginal zone before entering the red pulp and activating α7 nAChR expressed on PIC-secreting macrophages and dendritic cells which then suppresses the release of these and other pro-inflammatory molecules

The CNS responses to increased immune activity or the development of inflammation are largely mediated via neuronal and hormonal pathways. The neuronal route is mediated via the autonomic nervous system inflammatory reflex, whereby stimulation of the vagus nerve as an example leads to cholinergic interaction with the α7 subunit of the nicotinic acetylcholine surface receptor (α7 nAChR) of splenic macrophages, in turn leading to inhibition of inflammatory cytokine release by these T cells [107–109] (see Fig. 1). Neuroendocrine signalling between the immune system and the CNS is mediated by several discrete axes of interactions such as the HPA, hypothalamic–pituitary–thyroid (HPT), hypothalamic–pituitary–gonadal (HPG) and hypothalamic–pituitary–growth hormone axes, with the HPA axis being the most important [106, 110].

### The Role of Peripheral Inflammation in the Activation of the HPA Axis

Inflammatory signals can reach the brain via humoral and neural routes to activate the HPA axis [5]. The humoral route involves direct or indirect cytokine signalling either via direct access to the brain via regions where the integrity of the blood-brain barrier (BBB) is compromised or absent, such as the choroid plexus or other circumventricular organs (CVOs) [37], or by direct entry via saturable transport systems in the BBB or an indirect induction of cytokines and other inflammatory mediators such as prostaglandins and their subsequent release into the CNS parenchyma, or via provocation of an increase in BBB permeability [111, 112]. The neural route involves direct stimulatory action of PICs on peripheral afferent neurones of the vagus nerve [113, 114]. A broad array of cytokines such as TNF-α, IL-1, IL-6, IL-2 and IFN-γ can induce and regulate the HPA axis and, as will be discussed, their levels in turn are also heavily influenced by secretion of GCs [104].

### Mechanisms Involved in the Regulation of HPA Axis Activity

In physiological conditions, HPA axis activity is regulated by a multitude of afferent parasympathetic, sympathetic and limbic circuits such as the hippocampus, amygdala and medial prefrontal cortex which innervate the paraventricular nucleus (PVN) of the hypothalamus via a number of direct or indirect routes (reviewed in [97]). The PVN in turn integrates a number of stimulatory serotonergic, glutamatergic and catecholaminergic and inhibitory GABAergic signals, thereby representing a crucial hub in the regulation of HPA axis activity [115–117]. The activation of the HPA axis occurs following the indirect or direct stimulation of secretory neurones in the medial parvocellular region of the PVN or via the reduction of inhibitory inputs. The increased activity of these neurones leads to the release of corticotrophin-releasing hormone (CRH) and arginine vasopressin (AVP) into, respectively, the portal circulations of the anterior and posterior pituitary gland [7]. CRH in turn provokes the release of adrenocorticotrophic hormone (ACTH) into the systemic circulation by pituitary corticotropes. ACTH then ultimately induces the synthesis and secretion of GCs mainly by the zona fasciculata, and to a lesser extent by the zona reticularis, of the suprarenal (adrenal) cortex, followed by their systemic release [118, 119].

### Mechanisms Underpinning the Anti-inflammatory Responses of HPA Axis Activation

The release of GCs results in termination of HPA axis activation, in the absence of pathology, based on negative feedback achieved by the action of GCs on two main receptors, namely the cytosolic GR and the mineralocorticoid receptor (MR) which translocate to the nucleus following ligation [120, 121]. The MR has been described as a “promiscuous receptor” as, in humans, it binds to the mineralocorticoids aldosterone and 11-deoxycorticosterone, the GCs cortisol, 11-deoxycortisol and corticosterone, and the sex hormone progesterone (reviewed in [7]). MRs have a far greater affinity for GCs than do GRs (some ten times higher) [122] and are considered to be the principal regulator of circadian cortisol levels [123]. GRs, on the other hand, are considered to play a major role in regulating peak morning cortisol levels owing to their high affinity for dexamethasone and, crucially from the perspective of this paper, their role in anti-inflammatory and stress responses (reviewed in [124]).

Binding of a GC to a GR leads to the dissociation of molecular chaperones from the GR. The GC-GR complex then translocates to the cell nucleus where gene expression is regulated by interaction with glucocorticoid response elements and via protein–protein engagement with a range of other transcription factors, such as activator protein 1 (AP-1), nuclear factor-κB (NF-κB), nuclear factor of activated T cell (NFAT) and signal transducer and activator of transcription (STAT) [125–127] (see [128]). The main anti-inflammatory properties of GCs are mediated via the contact-driven suppression of NF-κB, AP-1 and STAT [129]. However, there is a mutually antagonistic relationship between GRs and these inflammatory transcription factors and there are data demonstrating that NF-κB, STAT and AP-1, which are elevated in inflammatory conditions, can in turn inhibit GR function [101]. The sensitivity of GRs to the action of GCs is also determined by a range of other factors, including GR function, GR number and GR affinity [129, 130].

## Mechanisms Underpinning the Association Between SED, ACE and the Genesis of Peripheral Inflammation

Low SES and the resultant multiplicity of chronic stressors in childhood effect epigenetic changes, particularly in the methylation status of DNA, leading to the production of a pro-inflammatory phenotype in macrophages and in turn causing an exaggerated inflammatory response to the presence of microbial antigens such as bacterial lipopolysaccharide (LPS) in childhood and adulthood [31, 131, 132]. There is also a growing body of evidence suggesting that chronic stressors stemming from SED affect the methylation status of GRs, accessory functional proteins and a range of genes regulating HPA axis activity [1, 31] (reviewed in [133]). Miller and fellow workers reported that exposure to chronic stress and associated changes in methylation lead to a decrease in transcription of proteins involved in GR signalling and an increase in the expression of transcripts with response elements for NF-κB governing pro-inflammatory signalling in monocytes [134]. Other research teams have reported very similar findings [135, 136]. Miller and colleagues reported an increase in monocyte pro-inflammatory activity but reported no changes in GR function or sensitivity, although they also noted a decrease in the transcription of proteins enabling GR function [137]. Changes in gene methylation patterns by chronic GC exposure are well documented (reviewed in [138]), but there is considerable debate regarding the persistence of environmentally induced epigenetic changes over a lifespan (reviewed in [139]). There is also evidence that the methylation status of a GR promoter gene is confounded by adult experiences [140]. In addition, there are a number of prospective studies demonstrating that the association between low economic status and significantly increased susceptibility to immune- or inflammation-mediated diseases is a result of persistent immune dysregulation [141, 142]. On the other hand, while compromised GR function could certainly play a role, a study of chronic stress in the caregivers of family members suffering from the aggressive brain tumour glioblastoma multiforme did not show any significant change in GR functioning compared with control subjects who were assessed as not having experienced major stressors during the previous year [137]. The question arises however as to how such immune dysregulation provoked by extreme childhood stressors persists into adulthood and why such a universally observed phenomenon leads to the development of pathology in some individuals but not others. We now move on to suggest a mechanism which might underpin this phenomenon based on individual genetic and epigenetic variance.

## Factors Involved in Influencing the Duration and Magnitude of an Inflammatory Response

### Genetic Factors

Several research teams have adduced evidence demonstrating that genetic factors strongly influence the intensity and duration of the immune response and the levels of inflammation produced by the activation of enzyme systems such as mitogen activated protein (MAP) kinases and Janus kinase (JAK)/STAT [143–147]. The most common source of genetic variation examined in connection to this area is that of functional single nucleotide polymorphisms (SNPs) [145, 146]. For example, in a large study involving 700 participants, Li and colleagues [148] reported a strong impact of genetic hereditability on the levels of cytokine production by PMBCs including monocytes. Pertinently, there is a considerable body of evidence indicating that SNPs in the genes encoding IL-1, IL-6 and TNF-α, which are all known to be elevated in people suffering from the effects of SED, influence the performance of the immune system [149–151]. It would also seem worthy of note that the composition of the microbiota also determines the levels of cytokines produced by the activation of immune and inflammatory pathways [152]. This may be relevant as exposure to chronic stressors can provoke dysbiosis and increased gastrointestinal permeability, thus allowing the translocation of commensal antigens into the peripheral circulation and provoking immune activation via the activation of TLRs on macrophages and dendritic cells [153, 154].

The significance of these findings extend to the observation that prolonged or excessive activation of immune and inflammatory pathways can induce tissue damage, leading to the formation of immunogenic DAMPs [35, 36]. These may activate TLRs on APCs leading to increased activity and levels of NF-κB and further increases in PIC levels. Increased levels of PICs can lead to still further increases in NF-κB production [155] and increased levels of NF-κB can stimulate the production of yet higher levels of PICs as well as cyclooxygenase-2,5-lipoxygenase and inducible nitric oxide synthase (iNOS), thereby driving a cellular environment of chronic oxidative and nitrosative stress (reviewed in [156, 157]). Increasing levels of PICs together with increasing levels of ROS and reactive nitrogen species (RNS) can induce further damage to tissues and macromolecules leading to increased DAMP formation and TLR engagement, ultimately producing higher levels of PICs and free radical species [36]. Increased levels of PICs ROS, RNS and NF-κB can also combine to drive a self-sustaining cellular environment of chronic inflammation and oxidative stress by modulating epigenetic mechanisms, notably DNA methylation and histone acetylation, responsible for governing the performance of immune and inflammatory pathways during and after activation, as we will now discuss [158, 159].

### Epigenetic Factors

#### Effect of PICs, ROS, RNS and NF-κB on DNA Methylation Levels

DNA methylation levels of CpG islands (DNA sequences with a high frequency of CpG sites) in promoter regions regulates the transcription of cytokine-encoding genes either directly or via regulating the activity of transcription factors such as STAT-1, NF-κB and NFAT-1 [160, 161]. These in turn regulate the production of these inflammatory mediators in response to the activation of numerous cellular stimuli such as the activation of PRRs on APCs by invading pathogens, the presence of DAMPs or increased levels of oxidative stress [161, 162](reviewed in [163]). In general, hypomethylation and increases in the production of these transcription factors result in increased levels of PICs, but the same effect can result from hypermethylation and reduced activity of molecules which inhibit PIC production such as the suppressors of cytokine synthesis (SOCS) [164]. PICs in turn affect the methylation status of genes involved in the regulation of immune and inflammatory activity by the suppression of methyltransferases and methylases which govern levels of gene DNA methylation [165, 166]. For example, high levels of PICs suppress the activity of DNA methyltransferase 1 (DNMT-1) leading to hypomethylation of the IL-1B promoter and a hundredfold increase in the serum levels of this cytokine [166]. DNA methylation levels also determine the magnitude and duration of immune and inflammatory responses by regulating the activity of macrophages and dendritic cells as well as regulating the activation and differentiation patterns of T cells [167, 168]. This is a brief outline of the interdependency between levels of gene methylation and the activity of immune and inflammatory pathways and readers interested in more details are invited to consult an excellent review by [169]. Increased levels of PIC production, ROS and RNS can cause changes in histone acetylation patterns in promoter regions of genes governing inflammatory signalling, which may also contribute to maintaining, if not amplifying, inflammation and oxidative stress via a positive feedback loop which we will now discuss.

#### Effects of Increased PICs, ROS, RNS and NF-κB on Histone Acetylation

Increased levels of inflammation, in the guise of elevated TNF-α and ROS, induce widespread hypomethylation and activation of a wide range of inflammatory transcription factors including NF-κB [170, 171]. Oxidative stress also induces hyperacetylation of NF-κB leading to increased DNA binding capacity [32]. Increased levels and transcriptional activity of NF-κB can induce the expression of co-inflammatory mediators via the activation of intrinsic histone acetyl transferase (HAT) activity and inhibition of histone deacetylases (HDACs) [170]. This is part of the complex effects of NF-κB on chromatin remodelling which drive the expression of genes and other transcription factors involved in regulating a plethora of inflammatory pathways [172, 173]. The activation of NF-κB also results in increased production of PICs and ROS, once again potentially contributing to the development of self-sustaining inflammation and oxidative stress [36].

#### Effect of PICs, ROS, RNS and NF-κB on miRNA Activity

miRNAs are small non-coding RNA molecules with roles in post-transcriptional regulation of gene expression and RNA silencing. Several miRNAs are induced by PICs, ROS, RNS and NF-κB [33, 174]. Furthermore, the weight of evidence indicates that the activities of PICs and miRNAs are strongly intertwined, as levels of miRNA expression change in response to stimulation by a range of cytokines, and cytokine gene expression is in turn regulated by changes in the activity of miRNAs and cytokine stimulation [175], while cytokine expression is regulated by miRNAs [176, 177]. Elevated levels of PICs and NF-κB can induce the production of microRNA-155 (miR-155) in macrophages, monocytes and myeloid dendritic cells [21, 176, 178]. Once activated, miR-155 increases the transcription of NF-κB, leading to increased production of PICs [179]. Similarly, NF-κB engages in a positive feedback loop with miR-181b-1 [179]. Briefly, NF-κB activation leads to increased levels of IL-6 which induces the phosphorylation of STAT-3 which transactivates miR-181b-1 which in turn transactivates NF-κB, providing yet another mechanism enabling self-sustaining levels of inflammation and oxidative stress [180, 181].

Another pro-inflammatory miRNA which is upregulated by PICs and NF-κB is miR-146a [21, 182, 183]. Unlike the case of miR-181b-1, however, the activation of this miRNA species does not lead to the transactivation of NF-κB but rather acts to inhibit this transcription factor via a somewhat complex negative feedback mechanism [179]. However, the upregulation of miR-146a has profound pro-inflammatory consequences and is involved in the development and propagation of neuroinflammation in illnesses such as MS and AD [184–186]. This miRNA was originally detected following LPS activation of monocytes but its greatest density is found in astrocytes and microglia. It might therefore be involved in the development and maintenance of low-grade neuroinflammation subsequent to the peripheral inflammation driven by prolonged SED and ACE [21, 187]. Given the information above, it seems reasonable to suggest that there are mechanisms which could at least partially explain how the activation of immune and inflammatory pathways in an individual exposed to SED or profound ACE could become chronic. We now move on to consider how genetics and epigenetics, chronic inflammation, as well as nitrosative and oxidative stress, could affect the activity of the HPA axis in childhood after showing that this effect could persist into adulthood.

## Factors Involved in Regulating HPA Axis Activity

### Genetic Factors

In adults, the inflammatory response to socioeconomic and other stressors is determined by genetic and epigenetic factors, with GR polymorphisms and GR methylation status both being involved [188]. GR and MR polymorphisms also play an important role in determining the magnitude and duration of HPA responses to the presence of environmental or internal stressors [189–191]. GR polymorphisms are associated with an increased risk of developing Crohn’s disease [192], metabolic syndrome, cardiovascular disease, neurological disease and neuroprogressive illnesses such as MDD [193–195]. Interestingly, GR polymorphisms are predictive of a significantly increased risk of developing post-traumatic stress disorder (PTSD) independently of childhood trauma. Polymorphisms in genes encoding proteins involved in regulating GC availability, such as the multidrug resistance transporter complex, 11 beta-hydroxysteroid dehydrogenase and corticosteroid-binding globulin, are also associated with an increased risk of developing AD, type 2 diabetes mellitus and a number of other inflammatory illnesses [130, 196–200]. It is also interesting that the performance of each of these proteins can be stimulated by immune activation [199]. At the very least, these data suggest that the levels of inflammation and immune activation experienced by individuals following SED can be significantly influenced by variability in genes regulating the activity of the HPA axis. The situation is rendered even more complex by data revealing that epigenetic factors also regulate the activity of the HPA axis [139] and data discussed above suggesting that an environment of chronic inflammation and oxidative stress leads to dysregulation of DNA methylation, histone acetylation and miRNA production, which could also drive abnormalities in HPA axis function.

### Epigenetic Factors

A growing number of research teams have proposed that methylation of genes involved in the regulation of GR function or expression within the HPA axis might be an important vehicle enabling changes in GC regulation in response to prolonged SED and/or ACE [139, 201, 202]. Indeed, the association between SED, ACE and *NR3C1* or *FKBP5* methylation has been established in several recent studies and systematic reviews [139, 201, 203, 204]. However, thus far, only the methylation of *NR3C1*, *FKBP5* and *HSD11β2* has been examined in direct relation to an increased risk for the development of any human illness, with the vast bulk of research being focused on the methylation status of alternative promoter regions of *NR3C1* [139, 201, 202]. Therefore, the effects of SED or ACE on methylation levels and clinical outcomes of the *POMC*, *ACTH*, *ACTH-R*, *AVP*, *CRH*, *CRH-R1/2* or *CRH-BP* genes, which are all involved in the regulation of GC levels and activity by the HPA axis, are currently unknown [205]. There is also some doubt regarding the biological importance of the changes in methylation levels reported by the authors listed above. For example, Palma-Gudiel and colleagues reviewed 23 papers investigating methylation changes in the *NR3C1* gene induced by ACE and concluded that the absolute differences in methylation levels are slight, with the majority of authors reporting group differences of less than 5%, which is currently close to the limit of sensitivity of the methylation detection assays utilised [133, 206]. These are important findings as there is a considerable body of evidence indicating that changes in the overall methylation levels of any gene of 10% may not be biologically relevant [207, 208] and changes of 5% or less should be treated with extreme caution [209].

In addition, it has been suggested that slight changes in gene methylation levels can be the product of failure to stratify results to take account of confounding variables such as age, sex, current SES and cell tissue type [210, 211]. The latter point may be especially relevant as recorded changes in methylation changes may ultimately originate from a small percentage of the cellular population, often described as marginal cellular subsets, leading to insignificant transcriptional changes. This is of interest from the perspective of SED- and/or ACE-induced methylation changes and the ultimate effect on HPA axis functionality as authors have reported a lack of association between methylation of *FKBP5* or *NR3C1* and increases in protein levels [212] or changes in GC regulation [213]. In addition, the concept that SED and ACE can induce methylation changes in HPA axis genes in the absence of genetic predisposition is under challenge, as several research teams have reported that changes in *FKBP5* and *NR3C1* methylation in MDD and PTSD patients with a history of SED and or ACE are only seen in people with acknowledged risk genotypes, which does not seem to be the case with SED- and ACE-induced changes in the methylation status of genes involved in the activation and regulation of peripheral immune genes [206, 214–216]. Finally, a review of 32 studies investigating the association between SED and or ACE on HPA axis gene methylation patterns by Argentieri and colleagues concluded that the vast bulk of studies are of cross-sectional or case control design and thus incapable of determining whether the methylation changes might be a cause or a product of pathology, which is a significant observation as such changes could be induced by the presence of inflammatory mediators, as discussed above [205]. However, this area is currently the subject of intense research and debate and it would be unwise to reach any firm conclusions based on current data. Readers interested in an in-depth examination of data appertaining to small changes in the methylation of genes and possible changes in the cellular proteosome are invited to consult an excellent review by Leenen and others [213]. For the sake of completeness, it should be noted that the performance of the HPA axis is also regulated by levels of histone acetylation [217, 218] and the activity of several miRNAs, such as miR-18 and miR-124 [219, 220], which may be dysregulated following exposure to prolonged stress [220, 221]. There is little evidence regarding the persistence of these changes, however, or any evidence of an association with human diseases or any association with a history of SEDs or ACE, and hence, they would not seem to be relevant to this review.

### Chronic Inflammation

**Fig. 2:**
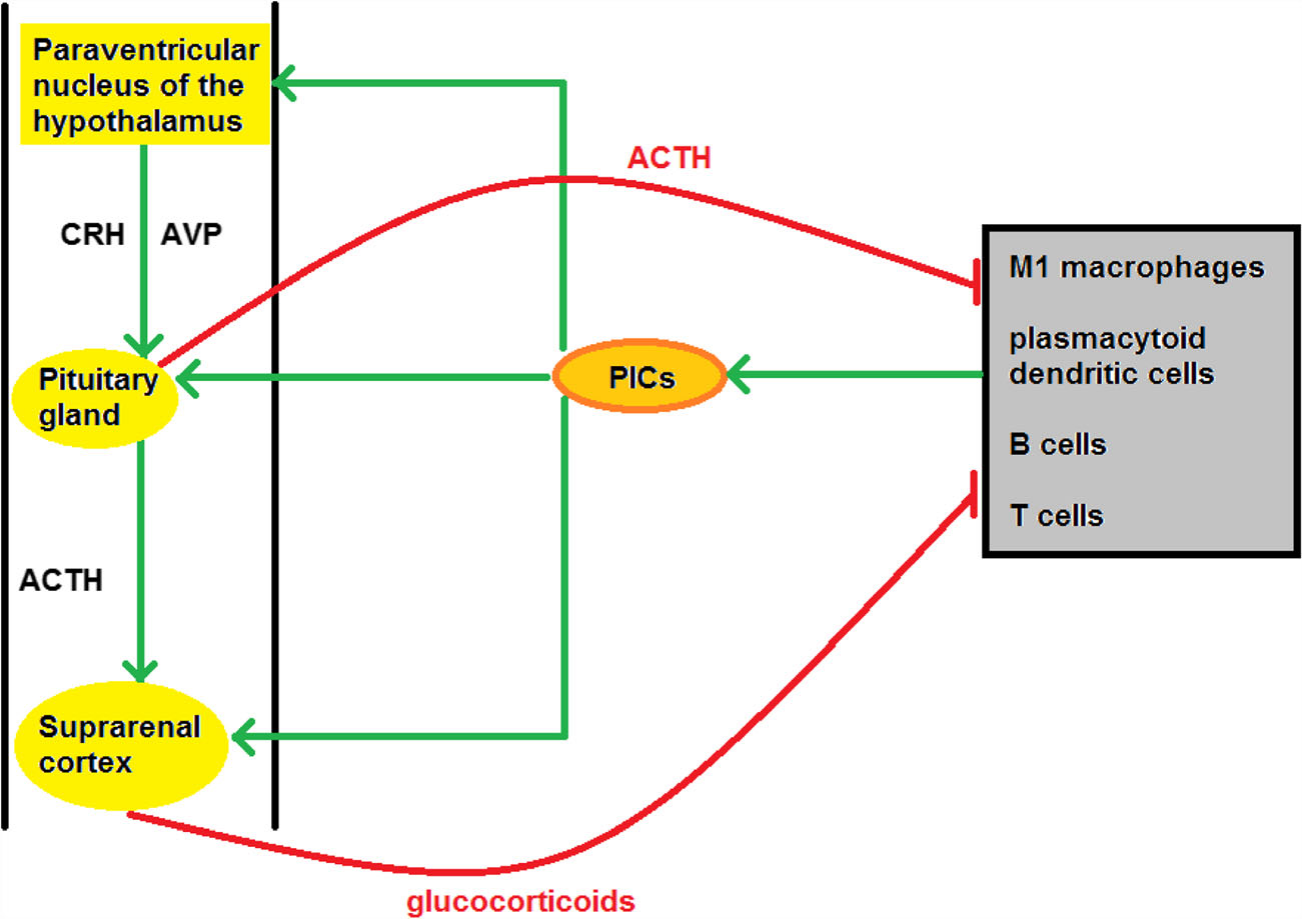
Bidirectional communication between the immune and neuroendocrine systems. PICs released by PMBCs can activate the HPA axis at the level of the pituitary, the paraventricular nucleus of the hypothalamus and the suprarenal (adrenal) cortex, stimulating the synthesis and secretion of GCs. The latter act on the surface or cytoplasmic receptors of PMBCs to suppress the transcription and translation of pro-inflammatory “Th1” cytokines such as IL-1 and IL-6 and increase the production of anti-inflammatory “Th2” cytokines such as IL-4 and IL-10, thus promoting a downwards shift in the immune response. ACTH release also exerts an independent direct immunosuppressive effect mediated via the melanocortin system

There is a large and accumulating body of evidence confirming the rapid downregulation of HPA axis activity in an environment of chronic systemic inflammation [222, 223]. This downregulation is largely mediated by PICs via a number of different mechanisms [224–226]. PICs such as TNF-α and IL-1β can inhibit the activity of CRH-secreting neurones and androgen synthesis in the suprarenal (adrenal) cortex, which may go some way to explaining the low basal levels of cortisol evident in several autoimmune, neurological and neuroprogressive illnesses [227, 228]. High levels of PICs also seem to have the capacity to attenuate stimulatory effects of CRH and ACTH on the suprarenal cortex and pituitary gland (see Fig. 2) [229]. Chronically elevated levels of PICs also appear responsible for HPA axis downregulation as a result of GR dysfunction leading to GC resistance. Cytokines such as TNF-α and IL-1β provoke GR dysfunction via several routes such as inhibiting the transcription of GRs and inhibiting their nuclear translocation [230]. There are also data indicating that IL-1β in particular inhibits GR function by activation of the p38/MAPK (MAP kinase) pathway [108].

A range of other enzymes and transcription factors upregulated by PICs such as JAK transcription factors, NFAT, AP-1 and NF-κB have the capacity to inhibit GR function [231, 232]. The mechanisms underpinning such inhibition include dysregulation or disruption of GR translocation to the nucleus, in a similar manner to the effect of PICs, and interference with GR-DNA binding via a series of protein–protein interactions. NF-κB, AP-1, IL-1, TNF-α, IL-6 and other inflammatory mediators such as cyclooxygenase (COX) can also disrupt the activity of crucial GR cofactors as well as provoke changes in the phosphorylation status of GRs [101, 233, 234]. Finally, there is also a body of evidence suggesting that chronic exposure to PICs changes the relative abundance of the two GR isoforms, with an increase in the dominant negative beta isoform and a decrease in the alpha isoform leading to a marked decrease in GR efficiency, which may be the major mechanism underpinning GC resistance in chronic inflammatory conditions such as RA [110, 235].

### Chronic Nitrosative and Oxidative Stress

An environment of chronic nitrosative and oxidative stress in the brain disrupts normal HPA axis function via several mechanisms including increased glutamate toxicity and reduced levels of RNA synthesis, RNA stability, mitochondrial function and activity of redox-sensitive kinases. This is a complex area and readers interested in a detailed treatment of this area are invited to consult the work of Spiers and others [236]. GC receptors are also under redox control and elevated concentrations of ROS exert detrimental effects on several aspects of GR function, such as translocation to the nucleus, leading to impaired feedback following HPA axis activation [237] (reviewed in [238]). High levels of nitrosative stress and consequently elevated levels of nitric oxide (NO) lead to a downregulation of GR expression [239, 240].

NO regulates a plethora of interactions between neuroimmune and neuroendocrine systems in physiologically normal and pathological conditions and neuronal nitric oxide synthase (nNOS) acts as a major modulator of processes governing the development of learning [241]. NO is involved in the regulation of corticosterone secretion and nNOS inhibits the activity of GR in the hippocampus resulting in the modulation of HPA axis activity [241, 242]. NO, in tandem with prostaglandins, plays a major role in enabling and regulating the activation of the HPA axis by CRH [243] (reviewed in [244]) and high NO levels exert broadly inhibitory effects [245]. Activation of the HPA axis leads to a profound inflammatory response and a significant increase in NO levels as well as increased levels of PICs and prostanoids [241] .

The downstream effects of NO and PICs are different however, and while CRH release, and consequent stimulation of ACTH secretion from the anterior pituitary, involves the synthesis and release of IL-1, IL-6 and TNF-α, NO released in the PVN modulates signal transduction pathways governing corticosterone release from the suprarenal (adrenal) gland [241]. NO also engages in “crosstalk” with GRs [246] and can upregulate these receptors to produce broadly anti-inflammatory effects [247]. However, at higher levels, NO inhibits GR binding [248, 249], offering another mechanism by which elevated nitrosative and oxidative stress compromises the anti-inflammatory capability of the HPA axis. In this context, it is noteworthy that loss of GR activity can produce aberrant GR-NO crosstalk leading to loss of neuroprotective functions conferred by astroglia in response to immune activation in the periphery [246]. There would appear to be ample evidence demonstrating that chronically upregulated levels of PICs, ROS and RNS can provoke dysfunction of the HPA axis. However, there is also evidence that stress-upregulated PICs, ROS and RNS in the periphery and the brain experienced during childhood can induce persistent and potentially pathological consequences in adulthood other than via detrimental effects on the neuroendocrine system, and we now turn to consideration of the evidence in this domain.

## Other Putative Pathological Consequences of Elevated PICs, RNS and ROS

### ACE and the Development of Oxidative Stress

Authors investigating the effects of social adversity in rodents have reported neuropathological consequences induced in part by increased levels of ROS and RNS coupled with reduced enzymatic and non-enzymatic anti-oxidants in the periphery and in areas of the brain such as the hippocampus forebrain striatum and cortex. Individual findings include reduced levels of superoxide dismutase (SOD) and catalase and widespread disruption of the glutathione (GSH) system such as a reduced GSH/GSSG ratio and depleted levels of glutathione peroxidase (GPx) [250–252]. These results are mirrored in human studies such as those examining the relationship between the extreme adverse social experiences during wartime relating to the development of neuropathology such as PTSD in general and Gulf War syndrome in particular, as we now go on to describe.

Once again, accumulating data indicate that increased brain oxidative stress stemming from war experiences is an important factor [253]. For example, excessive increases in cerebrospinal fluid (CSF) and CNS levels of NO/peroxynitrite is a reproducible finding in Gulf War veterans, whether or not such individuals suffered from PTSD, and appear to correlate with the extent of structural changes in the brain [253]. In addition, PTSD observed in American soldiers returning from Iraq has been associated with ROS-mediated brain changes [254]. In Gulf War veterans affected by PTSD, elevation in the levels of 8-hydroxy-2′-deoxyguanosine (8-OHdG) and the induction of 3-nitrotyrosine in the CSF have been reported; 8-OHdG is a biomarker of oxidative DNA damage [255]. Some research teams have also reported a putative association between the prolonged psychological stress of divorce and the development of significantly increased oxidative stress in the brains and periphery of adults and children [256]. This profile also appears to be dominated by a denuded cellular antioxidant system and thus reduced resistance to the corrosive effects of ROS and RNS [257].

The association between childhood sexual abuse and the development of persistent oxidative stress and inflammation is difficult to assess despite the existence of a plethora of studies investigating the subject. Some authors have reported an increase in both parameters in the short term but not in the long term while other authors have reported no difference compared with age- and sex-matched children with no history of such experiences [258, 259] (reviewed in [260]). The relationship between childhood maltreatment and the development of persistent oxidative stress appears to be much clearer however despite the difficulties in consistently defining the term [259, 261–263]. For example, do Prado and others reported that childhood maltreatment was associated with oxidative stress as shown by increased protein carbonylation, higher SOD levels and reduced GPx levels when compared with adolescents who had not undergone childhood maltreatment [261]. Very similar results have been reported in a more recent study by Morares and others who reported that various dimensions of childhood maltreatment such as physical abuse or neglect are associated with increased oxidative and nitrosative stress evidenced by the presence of lipid peroxidation, protein carbonylation and abnormalities in cellular enzymatic and non-enzymatic antioxidant levels [259]. These results have also been replicated by the same team of authors in an even more recent study [263]. In the latter instance, this research group also reported that the extent of lipid peroxidation and depleted antioxidant systems was predictive of the development and the severity of affective disorders which developed in later life [263].

The study conducted by Boeck and others is also of particular interest as these authors reported that childhood maltreatment was associated with increased oxidative stress as a result of increased mitochondrial ROS production secondary to increased activity in these organelles. Moreover, these authors also noted that such an elevation of ROS production and mitochondrial activity correlated positively with the release of PICs by PMBCs and depleted serum levels of lysophosphatidylcholines highly suggestive of increased levels of inflammasome activation [262].

### Oxidative Stress and the Development of Generalised Neuropathology

There is evidence to suggest that the source of oxidative stress seen in the brains of children and adults exposed to extreme and/or protracted life stress is activated NADPH oxidase (NOX) isoforms, most notably NOX-2 [264, 265]. This is perhaps unsurprising as NOX is upregulated in activated microglia and is the primary source of ROS production by these glial cells in this state [266, 267]. Once activated, NOX plays a major role in maintaining microglial activation and influences microglial polarisation towards the neurotoxic M1 phenotype [268].

The relationship between activated or ramified microglia and upregulated NOX-induced ROS production is of interest as severe or protracted psychological stress and the development of persistent peripheral inflammation known to activate microglia would go some way to explaining the relationship between severe life experiences and oxidative stress in the brain as discussed above [37, 111, 269, 270] (reviewed in [271]). It should be noted that stress-induced elevation of GCs in the brain increases microglial activation, priming and the development of a pro-inflammatory phenotype independently of the existence of peripheral inflammation [38–40]. Chronic and/or protracted stress also leads to “primed” microglia, which is a state which renders these cells exquisitely sensitive to further activation by even trivial peripheral stimuli such as minor infections or minor social stressors [272, 273]. This inherent sensitivity makes individuals more susceptible to the detrimental effects of “life experiences” and hence microglial priming could potentially provide a vehicle for conveying the effects of early-life stressors to adulthood and increases the chances of developing the neuropathology associated with neuropsychiatric and neurological conditions [274].

Early-life stressors may have a more direct effect on the later development of neuropathology, however, as the weight of evidence suggests that severe or protracted psychological stress may disrupt the normal bidirectional signalling between microglia, astrocytes and neurones with potentially devastating effects on brain function and brain development [275] (reviewed in [276]). Severe stress also has a direct and detrimental effect on levels of GABA, dopamine, serotonin, noradrenaline (norepinephrine) and glutamate neurotransmitters and various structures involved in neurotransmission mediated by each of these molecules, which could also play a role in communicating the effects of childhood psychological stress into adulthood (reviewed in [277]). For example, increased microglial ROS production mediates the development of glutamate toxicity, leading to mitochondrial damage and NMDA receptor upregulation, leading to further increases in ROS and the development of self-amplifying pathology [278, 279].

In addition, animal and human studies have demonstrated that psychological stress also increases glutamate levels and excitotoxicity in various regions of the brain including the prefrontal cortex, hippocampus and amygdala [280] (reviewed in [281]). Several research teams have reported that such changes in glutamatergic neurotransmission are induced directly by elevated GC levels [282, 283]. Activation of microglia and astrocytes via the existence of peripheral inflammation or increased levels of CNS GRs also has a detrimental effect of glutamatergic neurotransmission, as resting astrocytes play an indispensable role in glutamate uptake and the receptors that enable this activity are significantly downregulated or even absent on the membranes of these glial cells in the activated state [38–40, 111, 156]. Chronic glutamate upregulation also increases the expression of type 1 metabotropic glutamate receptors [284]. This is also of pathological relevance as upregulation and engagement of these receptors in the VPN inhibits the HPA axis stress response [285].

Finally, there is accumulating evidence from animal studies that genetic and epigenetic variation in the components of various neurotransmitter systems may go some distance to explaining the phenomenon of resilience (reviewed in [286]). For example, genetic variability in GABA receptors is associated with increased susceptibility or resilience to the effects of short term psychological stress while differences in the epigenetic regulation of serotonin receptors produces very similar consequences [287, 288]. This is clearly not the main focus of this paper and readers interested in the area are referred to an excellent and very recent review by Schiele and Domschke [289]. We now turn to the final section of the paper which examines mitochondrial dysfunction as vehicle for conveying multisystem dysregulation caused by ACE into adulthood.

## Mitochondria as Couriers of Childhood Stress into Adulthood

### ACE and the Development of Mitochondrial Dysfunction

A recent meta-analysis of 23 studies concluded that protracted and/or severe psychological stress is associated with profound and detrimental changes in mitochondrial performance, structural integrity, morphology and dynamics in the periphery and in the brain [290]. The most commonly reported indices of impaired mitochondrial function and respiration appear to be decreased complex I and IV activity, impaired complex I directed respiration, decreased membrane potential and increased permeability transition pore sensitivity [291–293]. Several research teams have also reported evidence of ultrastructural damage and altered morphology with increased lipid peroxidation, organelle swelling, loss of cristae and reduced matrix density [294, 295]. Psychological stress is also associated with reduced mitochondrial DNA (mtDNA) copy number in the rodent brain and changes in mtDNA transcription. For example, Liu and Zhou reported a 60% reduction in mtDNA copy number in corticosterone-treated rats in the hippocampus and striatum, with a slightly lower reduction in rats subjected to chronic unpredictable mild stress, while Roosevelt and others reported decreased transcription of mtDNA sequences coding for proteins involved in the structure of complex I of the electron transport chain (ETC) [291, 296]. Other authors have reported that psychosocial stress adversely influences global mitochondrial gene transcription [297, 298]. It is also noteworthy that there is some evidence that genetic variation in mtDNA may influence the susceptibility of mitochondria to the effects of psychological stress [299]. In addition, the use of metabolomics has revealed significant changes in the production and secretion of mitochondrial metabolites in the brain and blood of animals in a state of stress compared with unstressed controls [300, 301]. Furthermore, several authors utilising proteomic technology have reported strikingly similar results with significant changes in the protein composition of mitochondria during stressed versus unstressed conditions [302, 303]. The mechanisms whereby psychosocial stress exerts such a plethora of pathological effects on mitochondrial performance and physiology are the subject of intense research and may in part result from oxidative damage to mtDNA which is known to induce transcriptional abnormalities and organelle dysfunction [304, 305].

### Consequences of Mitochondrial Dysfunction

This is of prime importance as the weight of evidence suggests that variations in mtDNA sequences and/or expression influence the neuroendocrine, metabolic, inflammatory and transcriptional response to stress [15]. Moreover, mitochondrial dysfunction evidenced by dysregulated mtDNA expression or otherwise can have detrimental effects on the autonomic nervous system and the HPA axis as well as increasing levels of inflammation, lipids and glucose, which in total are regarded as being the main contributors to increased allostatic load and allostatic overload, which is predictive, at least to some extent, of the development of pathology as discussed above [14, 306] (reviewed in [16]).

The mechanisms whereby mitochondrial dysfunction can have detrimental effects on other harbingers of allostatic load are well documented and straightforward. For example, allostasis is an energy requiring process and environmental stressors provoke a significant increase in energy generation (reviewed in [290]). Similarly, mitochondrial dysfunction in adipocytes and insulin responsive tissues is a major, if not the main, driver of systemically elevated lipids and hyperglycaemia via well-established pathways (reviewed in [24, 25]). However, the mechanisms whereby mitochondrial dysfunction can have detrimental effects on the HPA axis and the neuroendocrine and transcriptional responses to stress appear to be under-discussed and hence are worthy of further consideration.

Epigenetic regulation of gene transcription, which plays a vital role in enabling allostasis as discussed above, is dependent on metabolites derived from the tricarboxylic acid cycle or produced elsewhere within mitochondria (reviewed in [23]). Importantly, this dependence relates to the addition and removal of epigenetic modifications. For example, cytoplasmic acetyl coenzyme A, derived from citrate exported from mitochondria, is an indispensable substrate enabling histone acetylation [307], and thus, at least a basal level of mitochondrial metabolism is needed to enable optimum histone acetylation [308]. Histone and DNA demethylation also depend on physiological levels of the citric acid cycle intermediate α-ketoglutarate and hence this process also requires optimum mitochondrial metabolism [309]. The precise relationship between compromised mitochondrial function and impaired genetic machinery is not fully understood but there is some evidence to suggest that it is facilitated by retrograde mitochondria to nucleus signalling [310], which also probably provides the vehicle whereby mitochondrial metabolism influences or regulates the expression of more than 50% of human genes [311].

Mitochondria are also the site of synthesis for GCs and all other steroid hormones and hence mitochondrial dysfunction could lead to significantly reduced GR production leading to a denuded anti-inflammatory response and impaired HPA axis feedback [312] (reviewed in [313]). GRs can also influence mitochondrial function following translocation into the organelles, probably via binding with GC response elements on mtDNA [314, 315]. Crucially, the weight of evidence suggests that chronic and/or excessive GR exposure during severe or protracted psychological stress dysregulates mitochondrial Ca^2+^ homeostasis, decreases mitochondrial respiration, damages enzymes of the ETC, reduces membrane potential and increases mitochondrial ROS production [316–318]. The actions of other stress hormones, notably the catecholamines noradrenaline and adrenaline (epinephrine), can also have detrimental effects on mitochondrial function and integrity in the periphery and the brain by inducing deletions in mtDNA [319, 320]. Finally, and perhaps predictably, there are accumulating data suggesting that the other effector molecule of the autonomic nervous system response to stress, namely acetylcholine, can also induce significant damage to mitochondria in all body compartments via engagement with the nAChRs on the outer membranes of these organelles [321]. Such engagement appears to disturb mitochondrial calcium homeostasis by inhibiting voltage-dependent anion channel (VDAC)-regulated Ca^2+^ uptake from the cytoplasm, opening the permeability transition pore resulting in the release of cytochrome *c* and activation of the PI3K/Akt signalling pathway [321, 322]. The sum of these effects can result in a potentially catastrophic fall in adenosine triphosphate production and, in the extreme, cell death via apoptosis or necrosis, with the latter having significant pro-inflammatory consequences [321, 322] (reviewed in [323]).

**Fig. 3:**
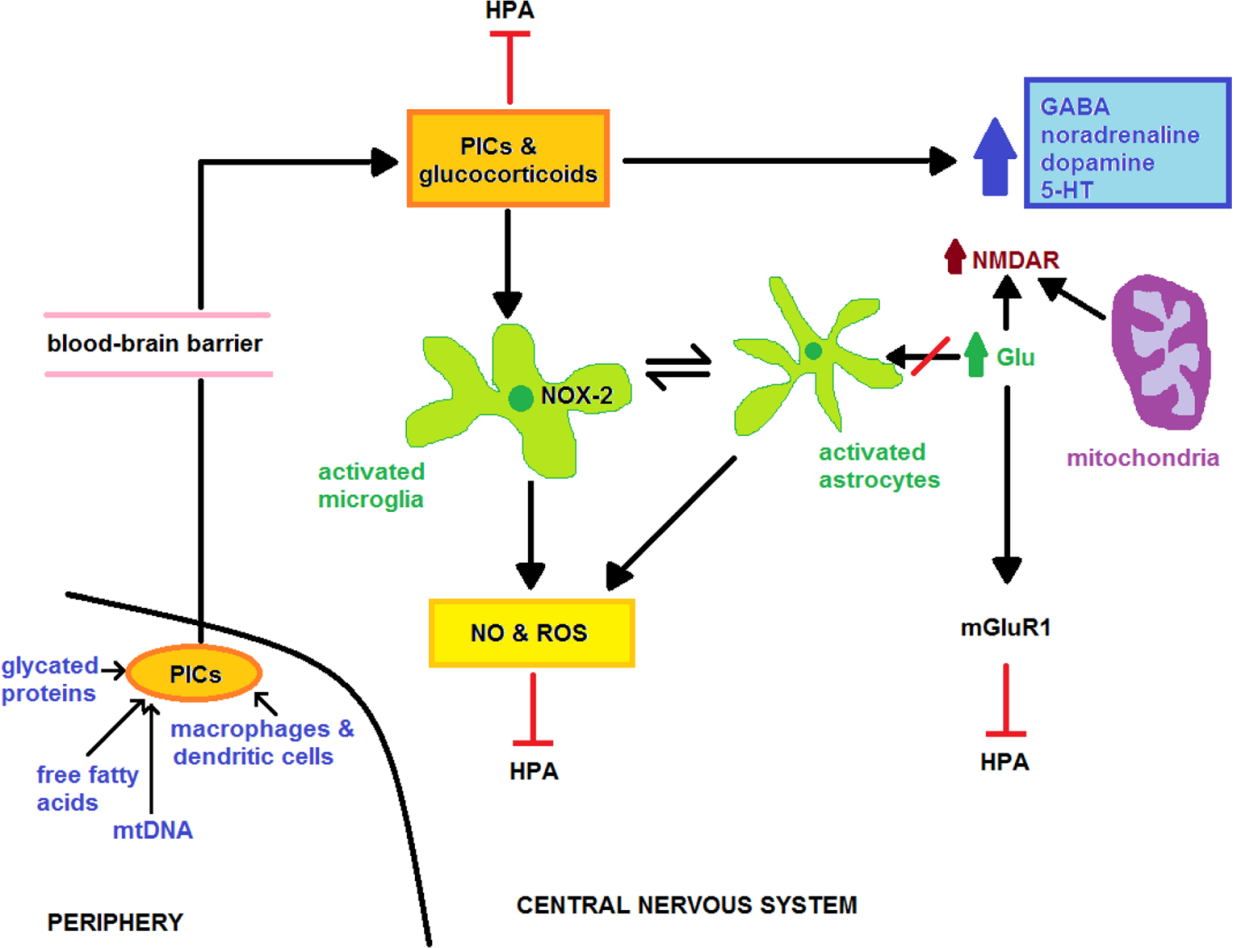
In this model, persistent peripheral inflammation caused by prolonged psychosocial stress in the guise of elevated PICs is transmitted to the brain via a number of well documented routes which then acts alone or in tandem with raised GCs to activate microglia and astrocytes. Their activation exerts a plethora of neurotoxic consequences such as the release of ROS, RNS and PICs and failure of astrocytic glutamate reuptake mechanisms, leading to glutamate excitotoxicity, increased activity of type 1 metabotropic glutamate receptors and NMDA receptor dysfunction. The ROS, RNS and PICs secreted by activated glial cells can also induce widespread dysregulation in GABAergic, serotoninergic, noradrenergic and dopaminergic neurotransmission as well as inhibiting the HPA axis via several mechanisms. Microglia and astrocytes may also be activated by elevated CNS GCs and prolonged or severe psychosocial stress in the absence of peripheral inflammation; elevations of these molecules can independently produce the same detrimental effects on glutamate excitotoxicity, NMDA dysfunction and other neurotransmitter systems as glial cell-derived ROS RNS and PICs. The development and persistence of peripheral and central inflammation are influenced by genetic and epigenetic factors (see text)

This is of importance as research teams have reported that under conditions of psychological stress damaged or dysfunctional mitochondria can release mtDNA and other mitochondrial DAMPs into the cytoplasm and circulation, leading to the development of systemic inflammation, immune activation and inhibition of the HPA axis [324, 325]. The mechanisms involved stem from the fact that mtDNA is a potent DAMP (also known as mitochondrial alarmin), owing to its close resemblance to bacterial DNA, and hence is able to activate AIM2 (absent in melanoma 2), NLRP3 (NOD (nucleotide-binding oligomerisation domain), leucine-rich repeats and pyrin domain-containing protein 3) inflammasomes and TLR-9 and provoke a type 1 interferon response [326] reviewed in [327]. Moreover, the presence of mtDNA in the systemic circulation can contribute to the development of systemic oxidative stress and further exacerbate systemic inflammation via the activation of NF-κB and the subsequent increase in levels of ROS, RNS and PICs [328, 329]. There is also evidence to suggest that the inflammation and oxidative stress invoked by mtDNA can inflict further damage on mitochondria leading to a feedforward loop of increasing inflammation and oxidative stress [326]. Such a scenario has the potential to cause oxidative and peroxidative damage to proteins and lipids and to provoke conformational changes which can also render these molecules immunogenic and capable of activating PRRs, thereby providing another route for self-amplifying inflammation and oxidative stress [35, 36]. It should also be noted that increased systemic levels of saturated fatty acids and hyperglycaemia, which can both result from mitochondrial dysfunction, can also potentially make an independent contribution to the development of systemic inflammation and oxidative stress [27, 28, 330]. Mechanistically, this is achieved via the activation of TLR-4 by free fatty acids [27, 331] and RAGE (receptor(s) for advanced glycation end products) by glycated proteins [28, 332]. It is also noteworthy that systemic inflammation either induced by “escaped” mtDNA or otherwise is known to activate microglia and hence could also account for data demonstrating increased levels of oxidative stress in the brain of animals and individuals exposed to severe psychosocial stress [37, 111]. In addition, it would seem that the sensitivity of microglia to developing a primed phenotype is dependent on genetic and epigenetic mechanisms [333]. The mechanisms discussed above are represented in Fig. 3.

## Conclusion

We have shown that there is strong evidence that childhood SED and/or ACE is associated with the development of adult HPA axis dysfunction and neuropsychiatric, neurodegenerative and autoimmune illnesses. Important contributors to the allostatic load experience during childhood include mitochondrial dysfunction, nitrosative and oxidative stress and inflammation, which in turn affect the regulation of HPA axis activity, including via epigenetic factors. We have also seen how PICs, RNS and ROS can have other pathological, including neuropathological, consequences and how mitochondria can act as couriers of childhood stress into adulthood. The latter is of importance as changes in mtDNA sequences and/or expression can influence the endocrine, metabolic, inflammatory and transcriptional response to stress. These findings provide pointers to future research and to potential therapeutic interventions.
